# Development of an ultra low noise, miniature signal conditioning device for vestibular evoked response recordings

**DOI:** 10.1186/1475-925X-13-6

**Published:** 2014-01-27

**Authors:** Chathura L Kumaragamage, Brian J Lithgow, Zahra Moussavi

**Affiliations:** 1 The Department of Electrical and Computer Engineering, University of Manitoba, Winnipeg, Canada; 2 Monash Alfred Psychiatry Research Centre, Melbourne, Australia; 3 Riverview Health Centre, Winnipeg, Manitoba, Canada

**Keywords:** Active shielding, Bio-signal amplifier, Electrocochleography, Electrovestibulography, Parallel amplifier, Power line interference, RFI filtering, Right leg driver, Ultra-low noise

## Abstract

**Background:**

Inner ear evoked potentials are small amplitude (<1 μV_pk_) signals that require a low noise signal acquisition protocol for successful extraction; an existing such technique is Electrocochleography (ECOG). A novel variant of ECOG called Electrovestibulography (EVestG) is currently investigated by our group, which captures vestibular responses to a whole body tilt. The objective is to design and implement a bio-signal amplifier optimized for ECOG and EVestG, which will be superior in noise performance compared to low noise, general purpose devices available commercially.

**Method:**

A high gain configuration is required (>85 dB) for such small signal recordings; thus, background power line interference (PLI) can have adverse effects. Active electrode shielding and driven-right-leg circuitry optimized for EVestG/ECOG recordings were investigated for PLI suppression. A parallel pre-amplifier design approach was investigated to realize low voltage, and current noise figures for the bio-signal amplifier.

**Results:**

In comparison to the currently used device, PLI is significantly suppressed by the designed prototype (by >20 dB in specific test scenarios), and the prototype amplifier generated noise was measured to be 4.8

$$\mathrm{nV}/\sqrt{\mathrm{Hz}}$$

@ 1 kHz (0.45 μV_RMS_ with bandwidth 10 Hz-10 kHz), which is lower than the currently used device generated noise of 7.8

$$\mathrm{nV}/\sqrt{\mathrm{Hz}}$$

@ 1 kHz (0.76 μV_RMS_). A low noise (<1

$$\mathrm{nV}/\sqrt{\mathrm{Hz}}$$)

radio frequency interference filter was realized to minimize noise contribution from the pre-amplifier, while maintaining the required bandwidth in high impedance measurements. Validation of the prototype device was conducted for actual ECOG recordings on humans that showed an increase (*p < 0.05*) of ~5 dB in Signal-to-Noise ratio (SNR), and for EVestG recordings using a synthetic ear model that showed a ~4% improvement (*p < 0.01*) over the currently used amplifier.

**Conclusion:**

This paper presents the design and evaluation of an ultra-low noise and miniaturized bio-signal amplifier tailored for EVestG and ECOG. The increase in SNR for the implemented amplifier will reduce variability associated with bio-features extracted from such recordings; hence sensitivity and specificity measures associated with disease classification are expected to increase. Furthermore, immunity to PLI has enabled EVestG and ECOG recordings to be carried out in a non-shielded clinical environment.

## Introduction

**Figure 1:**
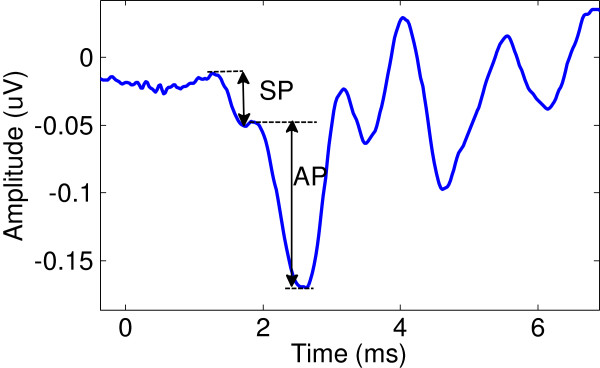
Typical ECOG response.

Development of low noise bio-signal acquisition devices have evolved substantially since the inception of the amplified electrocardiogram in the late 1940’s [1], due to the demand for high resolution electrophysiological measurements and increased patients’ safety [2]. One such method that demands a high resolution signal acquisition protocol is Electrocochleography (ECOG), a specialized technique that captures electrical activity from the cochlea, where the signal of interest can range from 0.1-2 μV for the extratympanic approach [3]. In this work the averaged electrocochleograph waveform is referred to as the ‘averaged field potential’ (AFP) that includes the summing potential (SP), and the whole nerve-action potential (AP) (see Figure 1). Bio-features identified in the AFP has enabled ECOG to be a useful clinical tool for diagnosis and monitoring of a balance disorder called Ménière’s Disease [3,4].

Our group is developing a novel technique called Electrovestibulography (EVestG) [5], a variant of ECOG, to be used as a diagnostic assistive tool for a subset of neurological disorders such as Depression, Parkinson’s Disease, and Schizophrenia [6,7]. In comparison to ECOG, EVestG captures electrical activity predominantly from the vestibular labyrinth during movements (which is analogues to the sound stimulus used in ECOG), while using a similar recoding topology (described in II). Research over the last decade shows that bio-features extracted from vestibular FP’s has potential to be used for classifying healthy subjects from patient groups [5]. The vestibular FP is assumed to be smaller (<1 μV_pk_) than the cochlea FP as the recorded response is not from stimulating the whole (or a tonal range of) auditory system in a synchronous response [3], rather, from detecting the spontaneous (or driven) response of smaller groups of “synchronously” firing otoacoustic hair cells. EVestG recordings are currently obtained using a commercially available, bench-top, low noise bio-signal amplifier in a sound attenuated electrically shielded chamber to reduce background interference. However, it is desired to implement a miniaturized ultra-low noise amplifier to reduce noise contributions from the recorder, and make EVestG a portable technology to be used in a clinical setting without the shielded chamber. The current apparatus has two major limitations. First, the system is susceptible to power line interference (PLI) at high gain (>85 dB) recordings, making it impossible to carry out recordings outside the shielded chamber. Second, the general purpose amplifier is not optimized for ECOG/EVestG recordings, hence limits high signal-to-noise-ratio (SNR) recordings that can be achieved if unique differences associated with the recording topology were identified and accounted for (detailed next).

Many groups have implemented low noise, bio-signal amplifiers with particular applications in neural recordings [8-13] using CMOS processes. Their common motivation was to develop the bio-signal amplifier to attain: low noise and low power characteristics, consume a small footprint, and to be integrated in multi-electrode systems. However, the referred to input (RTI) noise figure for these amplifiers [8-13] are >1.94 μV_RMS_ (for varying low pass cut off frequencies in the range 5 kHz to 9 kHz), and with varying mid-band gain values in the range 40-60 dB. For EVestG recordings we strictly require an ultra-low noise (<1 μV_RMS_) bio-signal amplifier with bandwidth 10 Hz-10 kHz, since amplifier generated noise ultimately dictates the lowest amplitude signal attainable. Furthermore, current noise generated by the amplifier is required to be small $\left(\sim \mathrm{fA}/\sqrt{\mathrm{Hz}}\;\mathrm{range}\right)$ to account for high input impedance scenario’s (not addressed in amplifier designs listed above).

## Background

ECOG/EVestG recordings are obtained by placing a specialized ear electrode proximal to the tympanic membrane (TM) via the ear canal, and a reference electrode on the earlobe. The difference between EVestG and ECOG recording topologies is that, the reference electrode is typically placed on the contralateral earlobe for ECOG [3], and on the ipsilateral earlobe for EVestG [5]. Noise associated with ECOG recordings are reduced by averaging FP’s resulting from auditory clicks, where all noise sources uncorrelated with the sound stimulus would diminish, leaving behind the AFP. EVestG in contrast, records spontaneous and driven vestibular FP’s, both at rest and when evoked by the vestibular stimulus through a whole body tilt [5]. These spontaneous FP’s occur at unknown times and time intervals, hence are extracted by a proprietary software algorithm called the Neural Event Extraction Routine (NEER) [14]. The algorithm’s FP detection accuracy is found to be largely dependent on three main types of interference present in the recording [15]: 1) biological signal interference within the bandwidth 40-500 Hz that is comprised predominantly of muscle activity [5]), 2) power line interference (PLI) (predominantly odd harmonics), and 3) system generated noise (where effects are significant for frequencies above 500 Hz).

**Figure 2:**
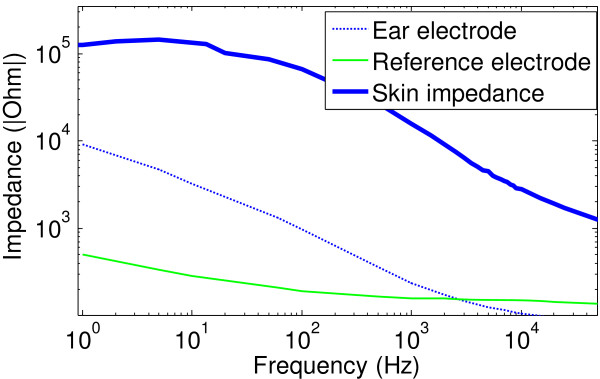
Impedance plots for the 2 electrode types used, and typical skin impedance measured from a test subject connected for EVestG recordings.

In common electrophysiological measurement techniques such as, electrocardiography (ECG), electromyography (EMG), and electroencephalography (EEG), typically identical electrodes are used for the differential electrode pair. In contrast the differential electrodes used for ECOG and EVestG are physically different, since the active electrode is a specialized one inserted in the ear canal, which is different from the reference electrode placed on the ear lobe. This mismatch of electrodes results in mismatched input impedances and cause common mode signals to be converted to differential mode, thus reducing CMRR of the system and increasing susceptibility to PLI [16,17]. As a preliminary step towards designing an appropriate amplifier configuration, the existing impedance mismatch was verified experimentally by immersing identical electrodes 1 cm apart in conductive gel filled in a 1 inch^3^ plastic cube. Figure 2 shows mismatched impedance values verses frequency for the two types of electrodes used; the solid green curve represents the reference electrode (ear lobe electrode), and the dashed line represents the ear electrode (each measurement was repeated 4 times and averaged; the worst case coefficient of variance (CV) for measurements was 5.2%), that would lead to large common mode signals. To attenuate common mode signals, successful digital filtering (post processing) approaches have been reported for ECG applications [18,19]. However, post processing alone does not suffice for large gain (>85 dB) applications, since saturation of the recording device can often occur due to PLI. Therefore, a driven right leg circuit (RLD) popularly used in electrophysiological measurements [1,16,18,20] was investigated in the prototype design (in section III) to minimize PLI from the source.

**Figure 3:**
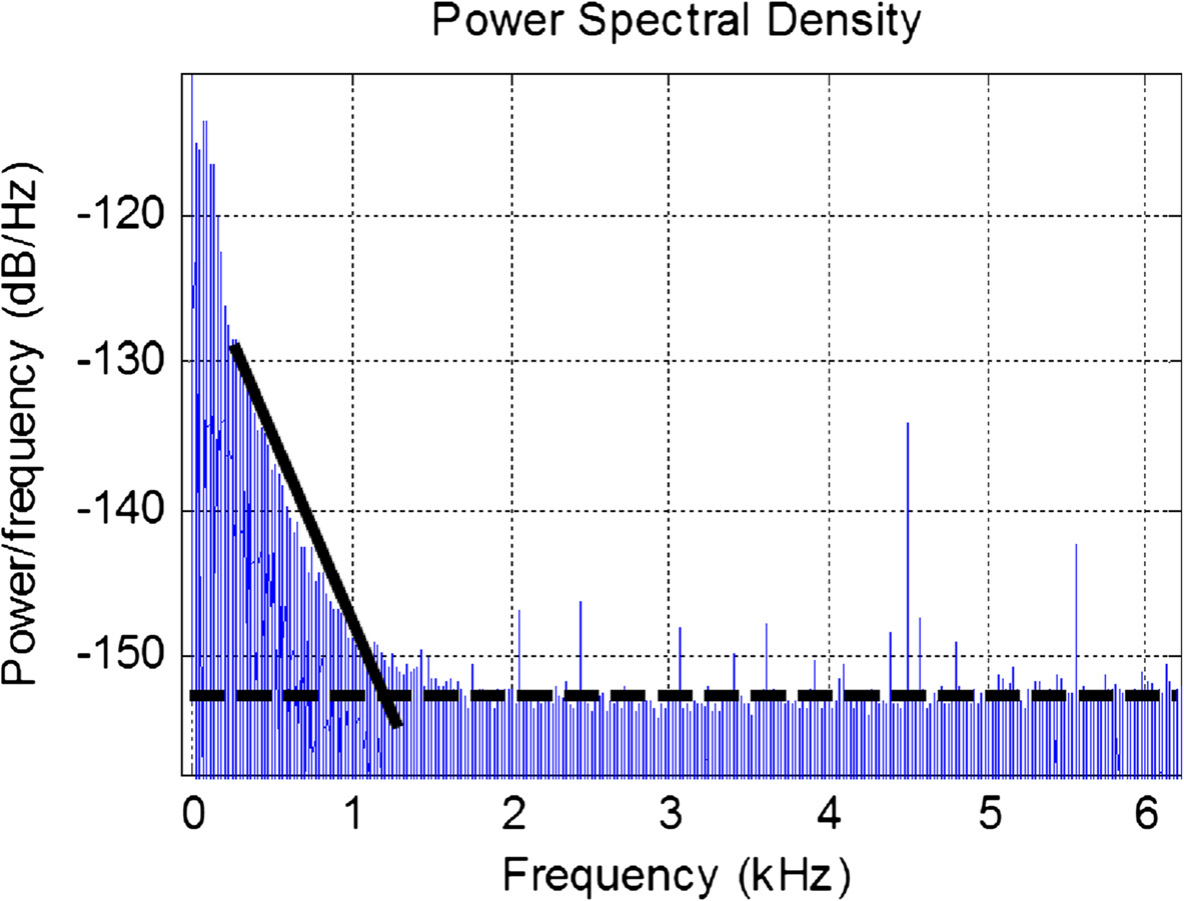
**Power spectrum density of a 5 min long ECOG recording.** The power axis is referenced to 1 Vrms.

Noise added by the recording apparatus (system noise) has a substantial effect on low amplitude recordings. Figure 3 illustrates a power spectrum of a 5 minute long ECOG recording, where the horizontal dashed line is indicative of system noise (a collection of noise generated by the amplifier, electrodes, and the electrode/skin interface [19,21,22]), and the low frequency (<1 kHz) component (solid line) corresponds to biological activity. The current OEM amplifier measures to have a 0 Ω load noise floor of $7.8\;\mathrm{nV}/\sqrt{\mathrm{Hz}}$ when tested at the connector box located approximately 1 m away from the device, which is also required to be minimized. Hence, detailed in this paper is the design, implementation, and validation of an ultra-low voltage and current noise amplifier tailored for ECOG and EVestG based on identified differences associated with recordings using commercially available components.

## Materials and methods

The proposed amplifier design consists of three major components to overcome the issues addressed above. They are:

1. Power-line Interference rejection using a driven right leg (RLD) topology

2. Active Shielding

3. Ultra-low noise Preamplifier design

In subsequent sections the requirements for these features are detailed, followed by validation testing.

A) The patient-amplifier interface model with RLD

**Figure 4:**
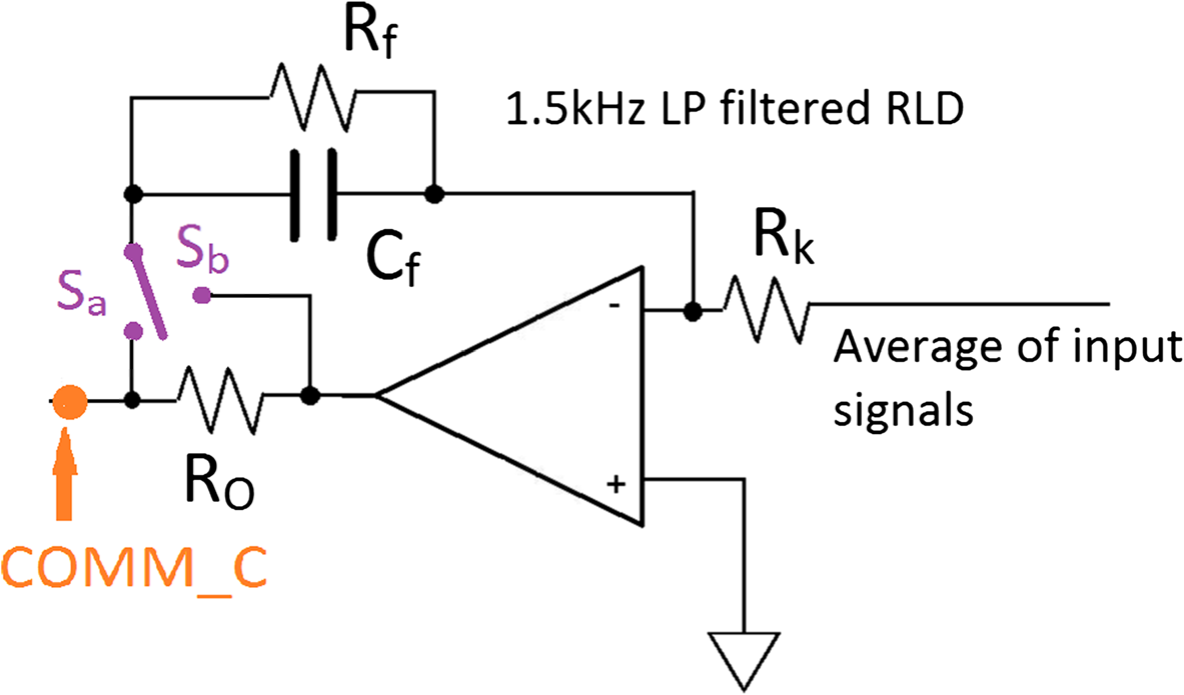
**Right leg driver circuit.** The cross section of the ear was adapted from [25] with permission.

**Figure 5:**
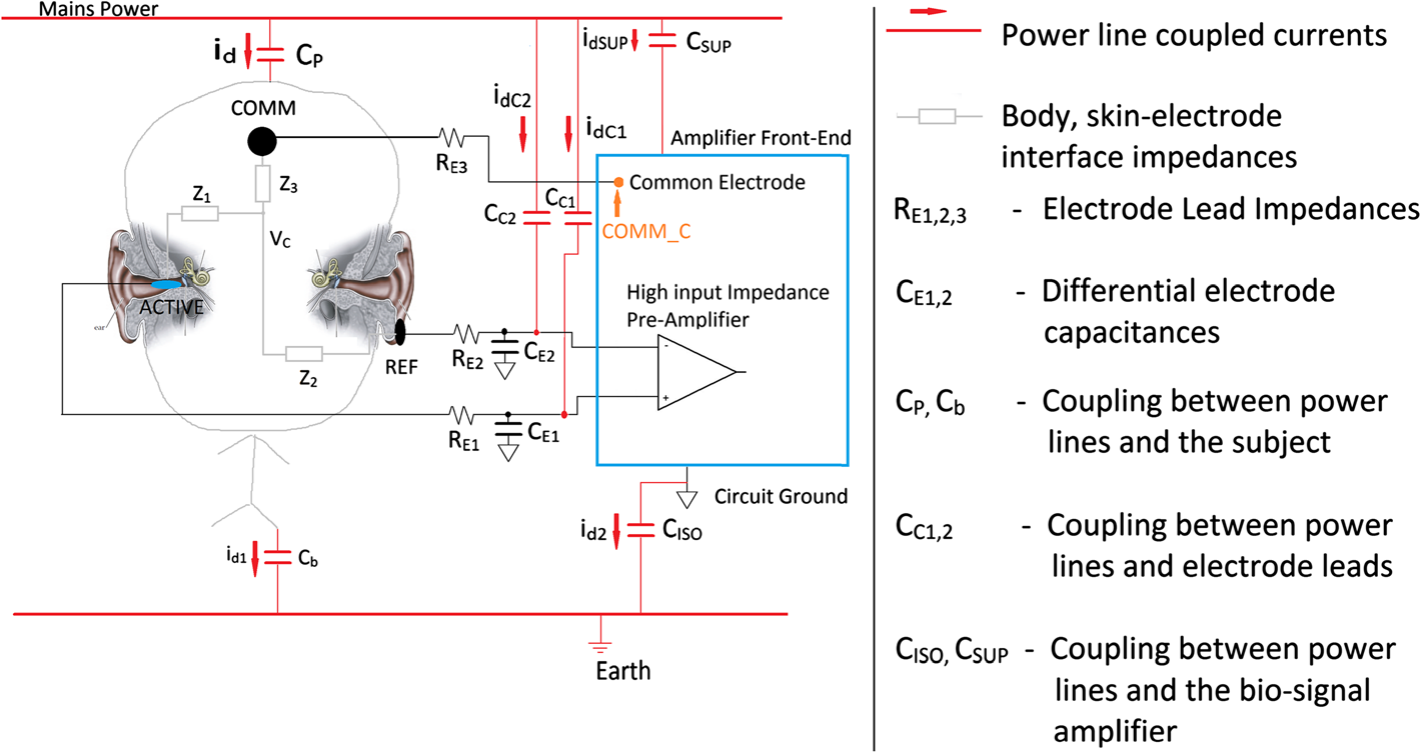
Circuit model for power line interference for ECOG recordings.

Figures 4 and 5 illustrates circuit models [16,20,23-25], when a person is connected with electrodes for an ECOG recording. Figure 5 shows inputs to the amplifier with intrinsic impedances from electrodes/leads and skin, and power line displacement currents and their pathways. For our scenario C_SUP_ (Figure 5) is negligible since the amplifier is battery powered. C_E1_ and C_E2,_ are parasitic capacitances, that arise due to the shield conductor around the differential electrodes; however, PLI coupling from C_C1_ and C_C2_ to the inner conductors are reduced due to this shield. By employing active driven shields, the parasitic capacitances C_E1_ and C_E2_ will be minimized (detailed in part III B), thus displacement currents i_dC1_, i_dC2_, and i_dSUP_ are negligible. However, it must be noted that neglecting these displacement currents do not affect the common mode voltage (V_C_) derived in subsequent equations, since it is based on the total displacement current *i*_*d*2_ as derived in (3).

Since displacement currents flowing in and out of the body must equate:

(1) $$i_{d}=i_{d1}+i_{d2}$$

where *i*_*d*2_ is flowing from the body to the common (COMM) electrode. The current division between C_ISO_ and C_b_ is used to express *i*_*d*2_ as follows:

(2) $$i_{d2}=\frac{C_{\mathrm{ISO}}}{C_{\mathrm{ISO}}+C_{b}}i_{d}$$

Then the common mode voltage V_C_ is

(3) $$V_{C}=i_{d2}\cdot Z_{\mathrm{RLD}}$$

where Z_RLD_ is the impedance between the V_C_ node and circuit ground. If the COMM electrode was connected to circuit ground, then:

(4a) $$Z_{\mathrm{RLD}}=Z_{3}+R_{E3}.$$

Figure 4 shows the COMM electrode driven by the RLD circuit (shown separately from Figure 5 for clarity). The RLD circuit consists of an inverting amplifier with gain $\left(G=\frac{R_{f}}{R_{k}}\right)$ with a corner frequency set at 1.5 kHz. The RLD circuit is driven by the signal common to both the differential electrodes (known as the common mode voltage). By driving the COMM electrode with an inverted *V*_*C*_ with gain (G + 1), the effective impedance *Z*_*RLD*_ simplifies to

(4b) $$Z_{\mathrm{RLD}}=\frac{\left(Z_{3}+R_{E3}\right)}{\left(G+1\right)}$$

From (2), (4b), and (3), V_C_ can then be expressed, for the S_a_ position in Figure 4, as:

(5a) $$V_{C}=i_{d2}\cdot \left(\frac{Z_{3}+R_{E3}}{G+1}\right)=\frac{C_{\mathrm{ISO}}}{C_{\mathrm{ISO}}+C_{b}}i_{d}\cdot \left(\frac{Z_{3}+R_{E3}}{G+1}\right)$$

For the case when the R_o_ resistor is outside the feedback loop (S_b_ position, see Figure 4), the impedance of R_o_ will also be added to the effective impedance Z_RLD_; hence

(5b) $$V_{C}=\frac{C_{\mathrm{ISO}}}{C_{\mathrm{ISO}}+C_{b}}i_{d}\cdot \left(\frac{Z_{3}+R_{E3}+R_{o}}{G+1}\right),$$

and from (2) and (3), if the 3rd electrode is grounded

(5c) $$V_{C}=\frac{C_{\mathrm{ISO}}}{C_{\mathrm{ISO}}+C_{b}}i_{d}\cdot \left(Z_{3}+R_{E3}\right).$$

From the three Equations (5a, b and c), it is seen that the lowest V_C_ value is attained from (5a) since the term R_0_ is not incorporated in the Equation.

B Active Shielding

**Figure 6:**
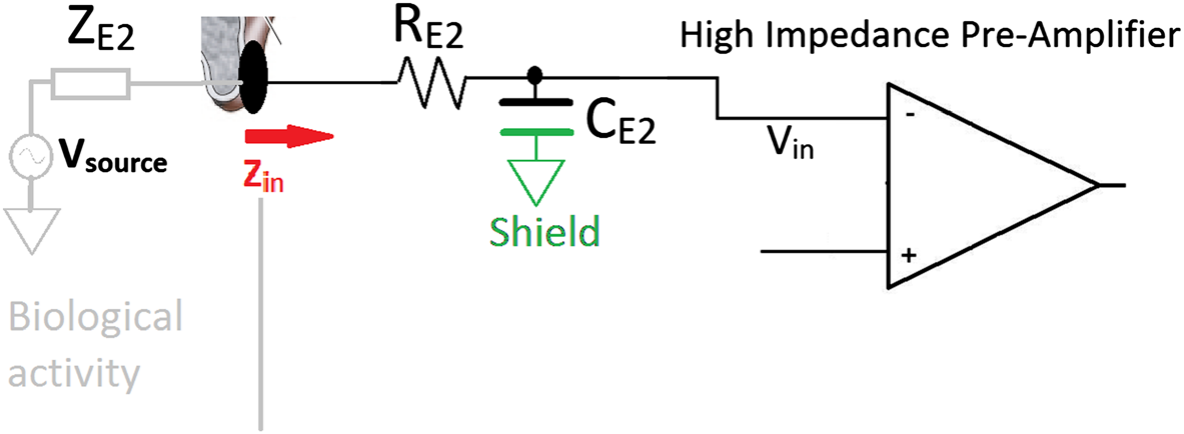
Electrical model of electrode Shield.

Shielded electrode leads are used to reduce PLI coupling [23,25,26]. Typically shield connectors are tied to ground, but results in parasitic capacitances (C_E1_ and C_E2_ of Figure 5) between the center conductor and shield (labeled as “Shield” in Figure 6). In high resolution, large bandwidth bio-signal recordings, this parasitic capacitance degrades the recorded signal due to two phenomena. First, with increased frequency the input impedance seen from the electrode (Z_in_) is reduced. Hence the signal amplitude at the amplifier input V_in_ attenuates due to voltage division shown in Equation (6), where *Z*_*in*_(*ω*) = *R*_*E*2_ + 1/*jωC*_*E*2_, and *Z*_*E2*_ is the impedance between the electrode surface contact and circuit ground.

(6) $$V_{\mathrm{in}}\left(\omega \right)=V_{\mathrm{source}}\cdot \frac{Z_{\mathrm{in}}\left(\omega \right)}{Z_{\mathrm{in}}\left(\omega \right)+Z_{E2}}$$

Secondly, the parasitic capacitances between the differential pair (C_E1_ and C_E2_) may differ (Figure 5), resulting in an impedance imbalance at the inputs and degrade CMRR at high frequencies.

Active shielding is therefore employed, which is derived from the input signal and drives the shield conductor. Driving the shield (using high speed op-amps with Gain-bandwidth-product of 18 MHz) with this signal maintains a near-zero potential across the lead conductor and shield. Therefore any current conducted by these capacitances (C_E1_, C_E2_) will be reduced; thereby contributing to high input impedance.

C Ultra-Low Noise Preamplifier Design

**Figure 7:**
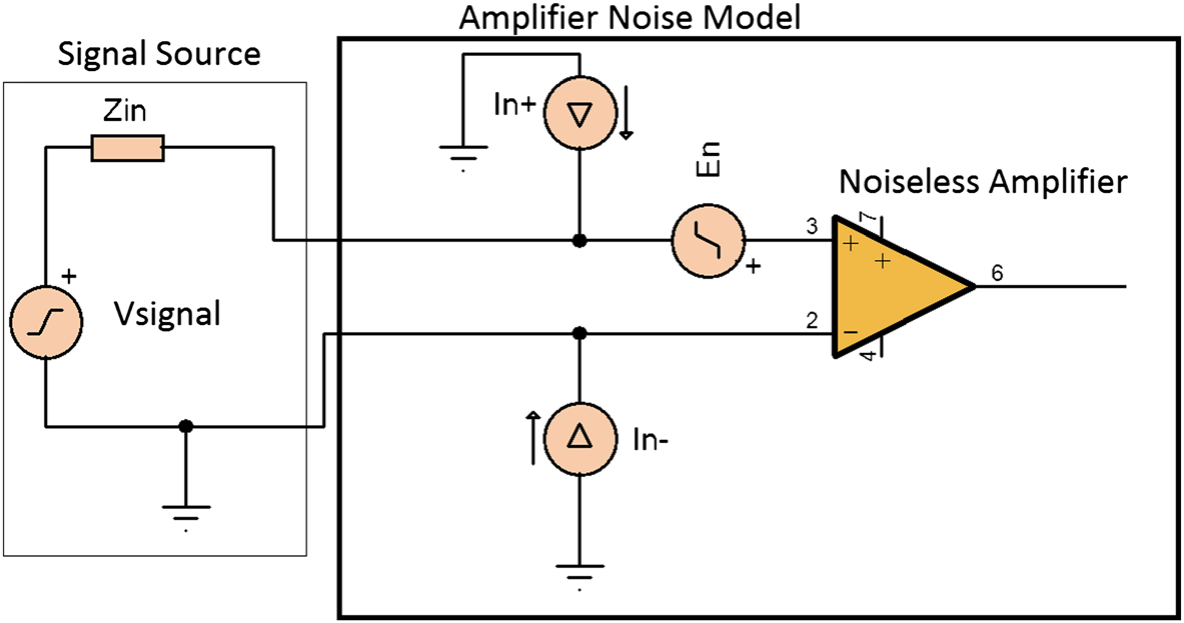
Amplifier noise model.

The design requirement was to implement a $<5\;\mathrm{nV}/\sqrt{\mathrm{Hz}}$ amplifier with very low current noise $\left(\sim \mathrm{fA}/\sqrt{\mathrm{Hz}}\;\mathrm{region}\right)$. The noise model of a generic amplifier can be represented as shown in Figure 7[27] (note that the noise sources shown represent voltage and current noise densities). Then the total RTI noise power spectrum density (PSD) Φ_total_ can be depicted as follows:

(7) $$\Phi_{\mathrm{total}}\left(f\right)=E_{n}^{2}\left(f\right)+E_{\mathrm{Zin}}^{2}\left(f\right)+|Z_{\mathrm{in}}^{2}\left(f\right)|\cdot I_{n}^{2}\left(f\right)$$

Here, the thermal noise of *Z*_*in*_ is EZin2f=4kBT⋅ReZinf[28] (*k*_*B*_ is the Boltzmann constant, and T is the temperature in Kelvin), and $|Z_{\mathrm{in}}^{2}\left(f\right)|\cdot I_{n}^{2}\left(f\right)$ is the voltage noise due to the current source in conjunction with the input impedance *Z*_*in*_. The noise component $E_{\mathrm{Zin}}^{2}\left(f\right)$ is contact dependent; the quantities $E_{n}^{2}\left(f\right)$ and $I_{n}^{2}\left(f\right)$ are voltage noise and current noise sources of the pre-amplifier model respectively. The RTI noise (RMS) value can then be calculated as follows:

(8) $$\mathrm{RTI}\;{\mathrm{Noise}}_{\mathrm{RMS}}=\sqrt{\int_{f_{1}}^{f_{2}}\Phi_{\mathrm{total}}\left(f\right)\;df}$$

In (8), f_1_ and f_2_ are high pass and low pass cut off frequencies of the analyzed signal respectively.

Currently available low noise operation amplifiers (OP amps) with bipolar transistors at the input stage can achieve very low voltage noise $\left(\sim 1\;\mathrm{nV}/\sqrt{\mathrm{Hz}}\right)$, at the cost of large current noise $\left(\sim \mathrm{pA}/\sqrt{\mathrm{Hz}}\;\mathrm{regime}\right)$[27], and precision FET input amplifiers can achieve very low current noise $\left(\sim \mathrm{fA}/\sqrt{\mathrm{Hz}}\;\mathrm{regime}\right)$, but with larger voltage noise $\left(>4\;\mathrm{nV}/\sqrt{\mathrm{Hz}}\right)$. A low current noise amplifier design is necessary in such bio-signal recordings since high input impedances (*Z*_*in*_) are common (see skin impedance plot in Figure 2). Therefore to achieve both low $E_{n}^{2}\left(f\right)$ and $I_{n}^{2}\left(f\right)$, a parallel amplifier approach inspired by [27,29] was attempted. If parallel amplification is used, the voltage noise introduced by the combined system will be reduced to $E_{n}/\sqrt{N}$ since the noise introduced by each amplifier is uncorrelated (N is the number of amplifiers in parallel).

However, there are consequences to parallelization that affect the performance of the overall amplifier. Three of them are: 1) the current noise will increase by $\;I_{n}\cdot \sqrt{N},$ 2) the input resistance reduces by $\frac{R_{\mathrm{in}}}{N}$, and 3) input capacitance increases by *C*_*in*_ ⋅ N. Maintaining input impedance as large as possible is necessary to minimize signal distortion due to loading effects [30]. In addition, the input capacitance increase reduces the overall amplifier bandwidth, where the low pass cut off frequency will be dictated by *f*_*LP*_ = 1/[2π(R_Load_ ⋅ C_in_)] (R_Load_ is the load resistance).

**Figure 8:**
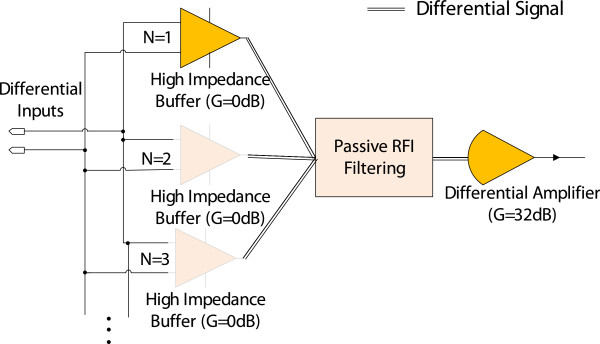
Parallel amplifier block diagram.

The designed preamplifier module (see Figure 8) is comprised of the following; a high impedance ultra-low current noise buffer stage $\left(G=0\;\mathrm{dB}\;\mathrm{and}\;\mathrm{En}=4\sqrt{2}\;\mathrm{nV}/\sqrt{\mathrm{Hz}}\right)$, a low noise RFI filter stage $\left(\mathrm{En}=1\;\mathrm{nV}/\sqrt{\mathrm{Hz}}\right)$, followed by a low voltage noise gain stage $\left(G=32\;\mathrm{dB}\;\mathrm{and}\;\mathrm{En}=1\;\mathrm{nV}/\sqrt{\mathrm{Hz}}\right)$ (detailed in the next subsection). It is advantageous to implement the input stage with gain >0 dB for improved noise performance; however, tolerance levels of resistor components are required to be in the 0.0001% regime to maintain >100 dB of CMRR [31]. As a compromise between noise performance and CMRR, a unity gain parallel pre amplifier approach was used, where the grayed out buffers are parallelized as shown in Figure 8. The simulated amplifier characteristics, with increased N, are summarized in Table 1. To investigate the contribution of current noise, Z_in_ = 1 MΩ is used to mimic poor skin contact. The thermal noise generated by the 1 MΩ resistance is calculated from the Nyquist equation to be $\sqrt{4k_{B}T\cdot \left[1M\Omega \right]}=129\;\mathrm{nV}/\sqrt{\mathrm{Hz}}.$ The total noise Φ_total_(1 *kHz*) is calculated from (7).

**Table 1:** Noise Performance with Increased Parallelization

	Φ_total_(1 *kHz*) 0 *Ω* load	Φ_total_(1 *kHz*) 1 M*Ω* load	**l**_**n**_	**C**_**in**_	**R**_**in**_	**Bandwidth for 1 MΩ load**
N = 1	$6.4\;\mathrm{nV}/\sqrt{\mathrm{Hz}}$	$129\;\mathrm{nV}/\sqrt{\mathrm{Hz}}$	$2\;\mathrm{fA}/\sqrt{\mathrm{Hz}}$	9 pF	~10 TΩ	17.7 kHz
**N = 2**	$4.9\;\mathrm{nV}/\sqrt{\mathrm{Hz}}$	$129\;\mathrm{nV}/\sqrt{\mathrm{Hz}}$	$2.8\;\mathrm{fA}/\sqrt{\mathrm{Hz}}$	**18 pF**	~**5 TΩ**	**8.8 kHz**
N = 3	$4.3\;\mathrm{nV}/\sqrt{\mathrm{Hz}}$	$129\;\mathrm{nV}/\sqrt{\mathrm{Hz}}$	$3.5\;\mathrm{fA}/\sqrt{\mathrm{Hz}}$	27 pF	~3 TΩ	5.9 kHz

For N = 1 to 3, Φ_total_(1 MΩ) is constant since the contribution of current noise is insignificant due to small I_n_. Note that Φ_total_(no load) for the case Z_in_ = 0 Ω does not decrease by the factor  because the entire pre-amplifier is not parallelized; only the buffered high input impedance stage is parallelized (Figure 8). In our design, for a load of 1 MΩ, the amplifier bandwidth decreases to 8.8 kHz for N = 2, and further reduces as N = 3. Hence increasing N beyond 2 was not considered. The implemented pre-amplifier had N = 2 with characteristics highlighted (bolded).

**Figure 9:**
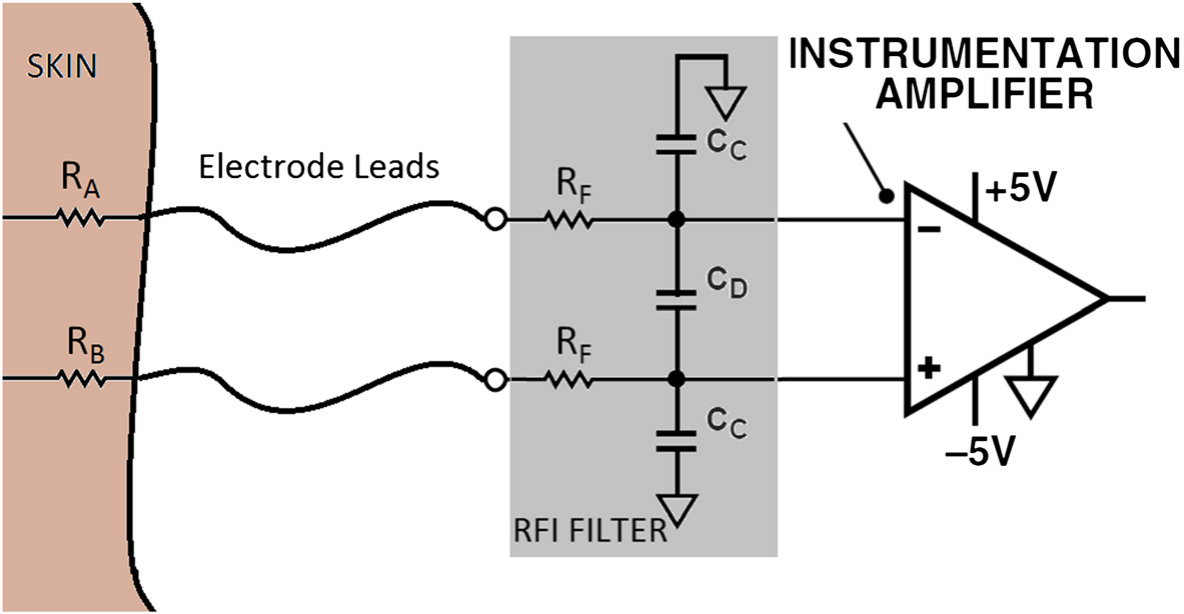
Traditional RFI filter network.

In bio-signal amplifier design applications RFI filtering is commonly employed at the input stage to attenuate HF content ahead of the instrumentation amplifier [32] (see Figure 9). RFI suppression minimizes DC output offset errors that can occur due to strong RF signal rectification in the instrumentation amplifier. The employed RC network is realized such that the common mode signal cut off frequency is higher than the differential mode cut off frequency as shown in (9a,b) [32], which minimizes CMRR degradation due to small variations in mismatched input impedances (R_A_ and R_B_).

(9a) $$F_{\mathrm{cutCM}}=\frac{1}{2{\mathrm{\pi RC}}_{c}}$$

(9b) $$F_{\mathrm{cutDiff}}=\frac{1}{2\mathrm{\pi R}\left(2C_{D}+C_{c}\right)}$$

where R is the skin impedance (R_A_ or R_B_) + R_F_

However, large mismatched differential inputs seen in ECOG/EVestG recordings (Figure 2) would lead to mismatched corner frequencies for the RFI filter if the traditional method is used; thus resulting in degraded CMRR and reduced signal bandwidth.

In commonly used bio-signal measurements, such as ECG/EEG/EMG, bandwidth reduction from the RFI filter due to large input impedances is negligible since the required signal bandwidth is <1 kHz. In contrast, the signal bandwidth of EVestG recordings spans up to 10 kHz, hence will be affected by bandwidth reduction. Secondly, with the traditional method (see Figure 9), C_D_ and C_C_ capacitors need to be in the ~ pF range to maintain high impedance at the electrode terminals, therefore large resistors (>5 kΩ) are required to realize the desired corner frequencies from (9a, b). The thermal noise of $\sim 9\;\mathrm{nV}/\sqrt{\mathrm{Hz}}$ from a 5 kΩ resistor is clearly unacceptable for this work, since the stringent low noise requirements wouldn’t be achievable if the traditional RFI filter network was employed. With the aid of the proposed RFI filter topology (see Figure 8) the following were achieved:

••Large mismatched input impedances will have no effect on the RFI corner frequencies.

••It is possible to set R_F_ at 56 Ω, that has an insignificantly small thermal noise $\left(0.96\;\mathrm{nV}/\sqrt{\mathrm{Hz}}\right)$ contribution, since there is no strict restriction on the values *C*_*D*_ and *C*_*C*_ to satisfy the required RC value. The capacitances *C*_*D*_ and *C*_*C*_ were therefore set at 1 nF and 5.6 nF respectively to obtain a ~200 kHz cut off frequency, while accounting for ~50 Ω output impedance on each side of the paralleled high impedance buffers.

D Overall Amplifier Design

**Figure 10:**
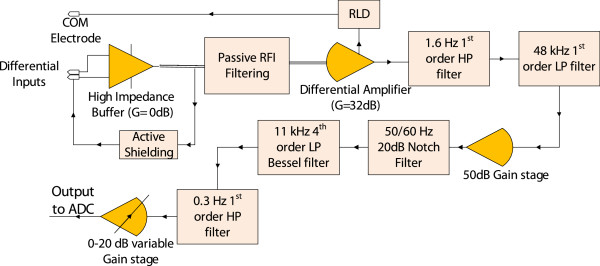
Overall circuit block diagram.

The overall amplifier block diagram, with the customized features described above, is shown in Figure 10. To maximize low noise performance, the pre-amplifier stage requires a large gain; however large DC offsets (~100 mV) often occur between the differential pair in bio-signal measurements due to the ‘half cell potential’ [30,33] that develops across the electrode and electrolyte interface. Hence a compromise between noise performance and stability must be met. After extensive test recordings, the pre-amplifier gain stage was set at 32 dB that maintained stability and low noise performance during recordings. After the pre-amplification, a 1^st^ order Butterworth high pass (HP) filter is employed to decouple frequency content below 1.6 Hz.

EVestG requires minimal phase distortion from the recording device, within the frequency band up to 9 kHz, since the NEER algorithm’s FP detection is based on phase changes across multiple scales obtained by a wavelet decomposition [5]. Hence an 11 kHz 4^th^ order Bessel Filter was employed as the primary low pass (LP) filter, which has maximally flat group delay characteristics.

Even though efforts were made to reduce power line harmonics with active shielding and driven right leg circuits, often large 50/60 Hz components are seen that can result in output saturation. Therefore a hardware notch filter at 50/60 Hz with a 20 dB depth and quality factor of 20 was employed. The output stage consists of a HP filter that decouples DC offsets introduces by active components in the circuit, and a variable gain stage (0 dB-20 dB). Table 2 summarizes parameters of the designed and implemented circuit.

**Table 2:** Overall circuit parameters

**Parameter**	**Measured**
**Input voltage noise density**	$4.8\;\mathrm{nV}/\sqrt{\mathrm{Hz}}$
**Input current noise density**	${}^{*}2.8\;\mathrm{fA}/\sqrt{\mathrm{Hz}}$
**Input Impedance**	*5 TΩ | 18 pF
**THD (@1 kHz, 0.2 V)**	0.0012%
**CMRR (@100Hz)**	115 dB
**Mid-band gain**	89 dB
**Bandwidth**	5 Hz-10 kHz
**Max input voltage swing**	±180 mV
**Power supply**	±9 V Batteries
**Total current**	64.2 mA
**Printed circuit board size**	9.9 cm X 5.4 cm
**Patient isolation**	*2 pF (from mains ground)

*Values obtained from datasheets and simulations.

E Experimental Setup

A CED 1401 analog-to-digital converter was used for all data acquisitions with sampling rate of 44 kHz. The prototype amplifier circuit ground was tied to the CED 1401 ground terminal, which was powered by a medical grade isolation transformer (POA MG240-1500-2-2010), thereby maintaining patient isolation from mains power. A CED-1902 amplifier with 1 Hz-10 kHz Bandwidth currently used for EVestG, was used to evaluate performance of the prototype amplifier.

Power-line interference analysis was performed on the RTI signal, and referenced to 1 V_RMS_ for computing the power spectrum in the dB scale. Noise figure comparisons are shown in two units: μV_RMS_: which is the RMS value of the recorded signal within the bandwidth 10 Hz-10 kHz, and with units $\mathrm{nV}/\sqrt{\mathrm{Hz}}$: which is the power spectral density value at 1 kHz.

F Electrocochleography (ECOG) Recording Setup

A conventional ECOG electrode setup was used [32], where a disposable TM-ECochGtrode (Bio-Logic) ear electrode is placed proximal to the TM, a reference electrode (ELS254S) placed on the contralateral earlobe, and the common electrode (ELS258S) placed on the forehead (Figure 5). A click stimulus was applied to the ipsilateral ear at 5.4 Hz with alternating polarity. The pulse width was set at 100 μs; with click intensity set to a loud but not uncomfortable level for the subject as it is common practice for ECOG recordings. Test subjects were volunteers (age 29 ±4, 3 males) with no record of hearing loss. Five minute recordings were taken while the subject was seated in a chair in an anechoic chamber. Ten millisecond windows of data from the start of each click (positive and negative separately) are extracted and averaged to reveal the AFP plot. Then, the action potential (AP) magnitude is measured (Figure 1).

G Electrovestibulography (EVestG) Validation Setup

Vestibular FP’s occur at unknown times and time intervals; hence qualitative analysis of the accuracy of captured FP’s from a human recording cannot be obtained for EVestG. One possible method of analysis is to obtain an additional trans-tympanic recording alongside the regular EVestG recording and compare the accuracy of captured FP’s. However, this would be an invasive approach. Instead, an artificial ear (middle ear and inner ear) [15] constructed to simulate electrical activity was used to compare the performance of the prototype amplifier for EVestG recordings. With the artificial ear, various sources of noise present in actual recordings can be applied at realistic proportions and be picked up by electrodes placed on the simulator. Cochlear AFP’s recorded in our recent work, and AFP shapes shown in [4,34] were used as a guide to generate the synthetic FP used. The FP detection accuracy of NEER was then evaluated for the prototype amplifier, and compared with that of the OEM amplifier in identical recording conditions.

## Experimental results

A Driven Right Leg Circuit (RLD)

**Figure 11:**
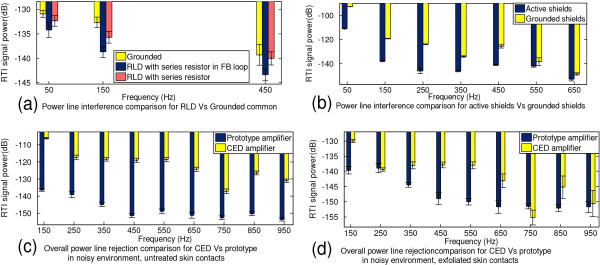
Power Line interference comparisons for RLD circuit (a), active shielding (b), and overall comparison of CED Vs prototype amplifier (c) and (d).

The prototype amplifier common (COMM) electrode is driven by the RLD circuit where R_f_ and C_f_ values set the LP corner frequency at 1.5 kHz, and $G=\frac{R_{f}}{R_{k}}=88$ was experimentally chosen since it produced the optimum CMRR from low frequencies (50 Hz) through to higher order harmonics (650 Hz) that are present in recordings. For ECOG recordings, the effect of PLI was tested for three cases of connecting the COMM electrode (see Figure 11a); RLD with switch on ‘S_a_’ position, RLD with switch on ‘S_b_’ position, and when the COMM electrode was grounded (node COMM_C in Figure 4). Recordings for each variant were 60s long, and were from the same subject obtained during one session with the same connected electrode leads. The prominent harmonics seen are the first harmonic (50 Hz), 3rd harmonic (150 Hz), and the 9th harmonic (450 Hz), hence the remaining harmonics were excluded from the figure. Results show that the RLD topology with the lowest impedance pathway (‘S_a_’ position in Figure 4) is the most effective at suppressing power-line interference, while both RLD topologies are better compared to the grounded case (5c). By employing an RLD topology *V*_*C*_ is attenuated for (5a) and (5b) in comparison to (5c), which increases overall CMRR of the system.

B Active Shielding

To evaluate PLI suppression as a result of active shielding, the RLD was bypassed, and the recordings were taken outside the anechoic chamber to enhance PLI. Five recordings of each case were obtained for 30 seconds and averaged. Power harmonics beyond 650 Hz are not visible since they are buried in the noise floor. We see that power line harmonics are reduced by at least 15 dB at frequencies (50 Hz, 150 Hz and 450 Hz) when active shielding was employed (Figure 11b).

C Overall Performance of the Proposed Amplifier in Comparison to OEM System

1. Power Line Interference Suppression

A large PLI scenario (that can occur during EVestG recordings) was mimicked by turning on the mains power driven lights inside the anechoic chamber, and a test subject was connected with electrodes for the EVestG topology. Sixty second recordings were taken from each amplifier for the same subject with no skin preparation to simulate poor contact, hence accounting for large impedances and impedance mismatches. The power spectrum results are shown in Figure 11c, where the prototype amplifier demonstrates PLI suppression of ~20 dB in comparison to the OEM amplifier (labeled CED) spanning up to 950 Hz. When the reference and common electrode sites were thoroughly exfoliated (Figure 11d), PLI suppression for both amplifiers is improved (due to reduced input impedance, hence reduced impedance mismatch), yet the prototype amplifier demonstrates better immunity as can be seen in Figure 11d.

1. Low Noise Performance

**Figure 12:**
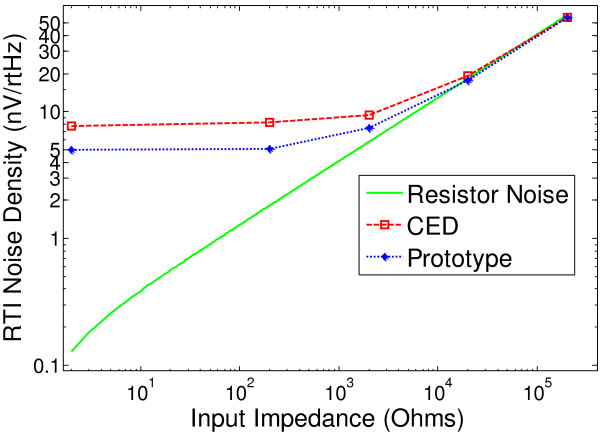
Prototype and existing OEM amplifier noise performance for varying input impedances, and the theoretical lower bound attainable (Resistor noise).

Noise performance of the CED amplifier and prototype amplifier were measured with respect to known input impedances (Figure 12). The prototype amplifier and CED amplifier were measured to have a “0 Ω load” noise floor of $4.8\;\mathrm{nV}/\sqrt{\mathrm{Hz}}\;\left(0.45\;µV_{\mathrm{RMS}}\right)$, and $7.8\;\mathrm{nV}/\sqrt{\mathrm{Hz}}\;\left(0.76\;µV_{\mathrm{RMS}}\right)$ respectively. The noise performance by impedances above 200 kΩ was not compared since the CED amplifier bandwidth is reduced below 1 kHz due to parasitic input capacitances at the input stage (see section III C).

1. ECOG Comparison

**Figure 13:**
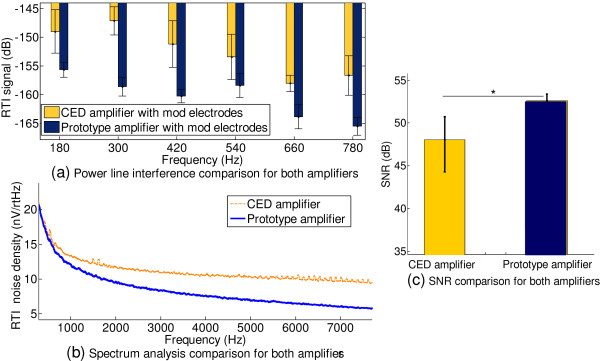
Comparison of PLI (a), power spectrum (b), and SNR for ECOG recordings (c) with the two amplifiers.

To evaluate SNR of the developed amplifier, ECOG recordings were obtained from a volunteer using both amplifiers in one session, while the sound stimulus was maintained at the same level. Two noise statistics were identified and compared: PLI (see Figure 13a), and the voltage noise spectrum (RTI) of the two recordings (Figure 13b). Towards low frequencies the curves overlap, which shows that the biological activity is similar in energy (as expected) for both recordings; however, beyond 1 kHz the spectral curves diverge. The CED plot plateaus at $\sim 10\;\mathrm{nV}/\sqrt{\mathrm{Hz}},$ while the prototype amplifier plateaus below $6\;\mathrm{nV}/\sqrt{\mathrm{Hz}}$ at high frequencies, which is indicative of the reduction in noise for the prototype amplifier due to the lower system noise. SNR calculations (where signal is the AP amplitude of the cochlear AFP) show that the prototype amplifier recordings were ~5 dB larger (*p < 0.05*) in comparison to recordings obtained from the CED amplifier.

1. EVestG Comparison

**Figure 14:**
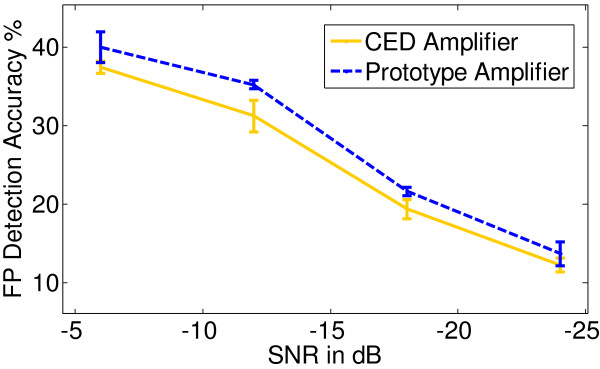
Prototype and OEM amplifier FP detection Vs SNR.

The prototype amplifier and CED amplifier were evaluated in the artificial gelatin setup [15], where the vestibular FP was buried in biological signal activity (EMG, ECG and EEG) at SNR values-6 to-30 dB in decrements of 6 dB. The biological signal energy was maintained at 5 μV_RMS_ that approximates background bio-signal activity captured in actual EVestG recordings. The performance of each amplifier is summarized in Figure 14, which illustrates the FP detection accuracy Vs SNR.

The prototype amplifier showed a mean increase in FP detection of 2.7 ± 1.1% compared to the CED amplifier *(p < 0.01)*. The FP amplitude-18 dB below background biological activity would be ~0.6 μV_rms_ in amplitude; in comparison, the prototype amplifier generated noise is 0.45 μV_RMS_ and the CED amplifier generated noise is 0.76 μV_RMS_, which is comparable to the FP amplitude. Therefore the overall increase in FP detection in the prototype can be attributed to the lower noise performance, and PLI suppression in comparison to the CED amplifier.

## Discussion

This work details the design and validation of an ultra-low noise bio-signal amplifier tailored for ECOG and EVestG. Recordings obtained from volunteers show that the RLD circuitry with active ground, and active shielding, effectively attenuates PLI in high bandwidth bio-signal measurements. PLI was shown to be suppressed more effectively in both shielded and non-shielded environments for the prototype amplifier compared to the OEM amplifier, which would enable recordings to be carried out in a non-shielded clinical setting; an essential component to making EVestG a portable technology. The RLD circuitry effectively reduces the COMM electrode impedance to circuit ground; yet the high level of isolation (~2 pF) between circuit ground and Earth ground limits the mains current flow through the leads (to <0.3 μA in the event that the live voltage 240 V, 50 Hz appears), and effectively protects the subject.

Implementing ultra low voltage noise amplifiers $\left(1\;\mathrm{nV}/\sqrt{\mathrm{Hz}}\right)$ is rather trivial given that on chip instrumentation amplifiers are available commercially; however, due to the large input impedances that can occur in EVestG/ECOG recordings, ultra-low current noise characteristics are also required. In an attempt to design the amplifier with voltage noise $<5\;\mathrm{nV}/\sqrt{\mathrm{Hz}}$ and current noise in the $\sim 2\;\mathrm{fA}/\sqrt{\mathrm{Hz}}\;\mathrm{range}$, a unique parallel pre-amplifier approach was investigated and validated. The traditional method of using RFI filtering cannot be employed in such low-noise applications due to the large series resistances required. The drawback of the implemented RFI filter topology is that, the buffered input stage is susceptible to RFI that could lead to DC offset errors from the differential pair. However repercussions due to this phenomenon were not seen throughout extensive recordings conducted.

In early stages of the design, the preamplifier was configured to have a large (40 dB) gain to maximize noise performance. The amplifier functioned as expected for EMG, ECG, and EOG (electrooculogram) recordings, however displayed instability during ECOG and EVestG recordings. This issue was identified to be caused by mismatched electrodes that lead to mismatched DC potentials (>180 mV) appearing on each electrode (a result of the electrodes’ half cell potential [30,33]), causing amplifier saturation. As a result, the preamplifier gain was reduced to 32 dB to avoid instability at the cost of a reduction in noise performance from $4.6\;\mathrm{nV}/\sqrt{\mathrm{Hz}}\;\mathrm{to}\;4.9\;\mathrm{nV}/\sqrt{\mathrm{Hz}}$ (in simulation). These mismatched DC potentials between leads indicate that separately driving each lead wire shield would be more effective in reducing parasitic capacitances C_E1_ and C_E2_. Therefore individual active shields were employed.

μV amplitude ECOG electrical activity obtained from the prototype amplifier show significantly higher (*p < 0.05*) SNR as a result of improved noise performance and improved PLI suppression; furthermore, FP detection accuracy of the NEER algorithm was also shown to increase (*p < 0.01*) for prototype amplifier recordings in identical test scenarios compared to the OEM amplifier (due to the reduction in system noise from $7.8\;\mathrm{nV}/\sqrt{\mathrm{Hz}}\;\mathrm{to}\;4.8\;\mathrm{nV}/\sqrt{\mathrm{Hz}}$). The increased FP detection accuracy has significant implications for EVestG; it will incur a reduction in variability associated with bio-features extracted, which is expected to result in increased sensitivity and specificity measures used for disease classification.

Table 3 compares key features of state-of-the-art (CMOS) low noise bio-signal amplifiers, the CED amplifier, and the prototype amplifier. CMOS devices listed, have noise performance $>16\;\mathrm{nV}/\sqrt{\mathrm{Hz}}$ (> 2-fold worse than the CED amplifier noise performance) which is inadequate for EVestG, since FP detection accuracy will be further degraded rendering the methodology ineffective for disease classification. However, for the increase in noise performance of ~5-fold, compared to CMOS devices, the prototype amplifier sacrifices power consumption and footprint requirements by factors ~10^4^ and ~5x10^4^ respectively. These substantially large power and footprint requirements however are tractable, since only two such channels are required for a complete EVestG system; in comparison, CMOS devices listed are for multi array (hundreds of channels) applications, which require low power and footprint characteristics per channel to accommodate the entire array. As a result of the high power and footprint requirements, implementation of an ultra-low noise, multi-channel recording device with the prototype amplifier is currently not feasible. However, the prototype amplifier has other applications in low noise recordings such as high resolution ECG [35]. An example would be ventricular tachycardia, a life threatening condition, described to have low amplitude and high frequency content at the end of the QRS complex and the ST segment [36]. Accurately extracting such features require a low noise, high bandwidth amplifier, such as the prototype device presented in this manuscript. Once the prototype amplifier is validated on a large (n > 20) patient group population, a miniaturized single chip design will be investigated in collaboration with Texas Instruments that will reduce power and footprint requirements.

**Table 3:** Performance comparisons against other systems

**Parameters**	[8]	[9]	[10]	[11]	[12]	[13]	**CED ampifier**	**This work**
Supply voltage/Current	±2.5 V/16 μA	±2.8 V/2.7 μA	1.8 V/4.7 μA	3.3 V/8 μA	1.8 V/4.4 μA	1.5 V	±15 V/ -	**±9 V/64.2 mA**
Gain	39.5 dB	40.8 dB	49.5 dB	39.6 dB	39.4 dB	10-62 dB	88.4 dB (configured)	**89 dB**
Bandwidth	7.2 kHz	45 Hz-5.3 kHz	9.1 kHz	8.2 kHz	7.2 kHz	10 kHz	10.1 kHz	**11 kHz**
Voltage noise density (RTI)	$\sim 26\mathrm{nV}/\sqrt{\mathrm{Hz}}$	$\sim 42\mathrm{nV}/\sqrt{\mathrm{Hz}}$	$\sim 58\mathrm{nV}/\sqrt{\mathrm{Hz}}$	$\sim 21.4\mathrm{nV}/\sqrt{\mathrm{Hz}}$	$\sim 41.2\mathrm{nV}/\sqrt{\mathrm{Hz}}$	$<16\mathrm{nV}/\sqrt{\mathrm{Hz}}$	$7.8\mathrm{nV}/\sqrt{\mathrm{Hz}}$	$4.8\mathrm{nV}/\sqrt{\mathrm{Hz}}$
CMRR	≥83 dB	≥66 dB	≥52.7 dB	≥76 dB	70.1 dB	>90.7 dB	117.6 dB (@100 Hz)	**115 dB (@100 Hz)**
Area	0.16 mm^2^	0.16 mm^2^	0.05 mm^2^	3 mm x 3 mm	0.06 mm^2^	0.06 mm^2^	24 cm X24cm	**9.9 cm X 5.4 cm**

Outcomes of this work and previous work [15] suggest that amplifier generated noise is only one aspect that contributes to degrading the quality of the recording. Noise due to electrodes and biological interference also need to be minimized to maximally utilize low noise characteristics of the designed prototype amplifier, which will be investigated to improve the quality of EVestG recordings.

## Conclusion

ECOG and EVestG are electrophysiological measurement techniques that record activity in the μV range, where susceptibility to various sources of noise severely degrade SNR. In this paper, an ultra-low noise, and miniaturized bio-signal amplifier tailored for vestibular and cochlear evoked potentials was designed and evaluated. Based on human recordings and test-bench evaluations conducted, the prototype amplifier demonstrated to surpass performance of the current OEM device, and state-of-the-art devices, in terms of PLI suppression and noise performance for ECOG and EVestG recordings.

## Abbreviations

AFP: Averaged field potential; AP: Action potential; EVestG: Electrovestibulography; CMRR: Common mode rejection ratio; COMM: Common/ground electrode; EEG: Electroencephalography; ECG: Electrocardiograph; ECOG: Electrocochleography; EMG: Electromyography; FP: Field potential; NEER: Neural event extraction routine; OEM: Original equipment manufacture; PLI: Power line interference; RLD: Right leg driver; RTI: Referred to input; SNR: Signal to noise ratio; SP: Summing potential; TM: Tympanic membrane.

## Competing interests

The authors declare that they have no competing interests.

## Authors’ contributions

CK designed, implemented, and validated the prototype amplifier presented in this work. He also conducted the data analysis and wrote this manuscript with guidance provided by BL and ZM. BL conducted the majority of signal recordings, he was involved in amplifier testing, and drafting and revising of this manuscript. ZM was involved with statistical analysis and data interpretation, and with the drafting and revising of this manuscript. All authors read and approved the final manuscript.

## Authors’ information

CK was a M.Sc student in biomedical engineering at the University of Manitoba (2010-2013). The work involved investigating a low noise signal acquisition protocol for a novel electrophysiological measurement technique called Electrovestibulography (EVestG). He is now a PhD student in Biomedical Engineering at the University of McGill.

BL is the inventor of EVestG technology. He is a senior research fellow at the Monash Alfred Psychiatry Research Centre (MAPrc) EVestG Lab, at the Alfred Hospital Melbourne Australia. He is an adjunct professor in electrical and computer systems engineering at University of Monash, an adjunct professor in electrical and computer engineering at the University of Manitoba, and a Research Affiliate at the Riverview Health Centre, Winnipeg Canada.

ZM is the director of the biomedical engineering program at the University of Manitoba. She is Canada Research Chair & Professor, Dept. of Elec & Comp. Engineering and Dept. of Psychiatry. She is also a Research Affiliate at the Riverview Health Centre, Winnipeg Canada.
